# A “3S+f” Nephrometry Score System to Predict the Clinical Outcomes of Laparoscopic Nephron-Sparing Surgery

**DOI:** 10.3389/fonc.2022.922082

**Published:** 2022-07-14

**Authors:** Shudong Zhang, Zijian Qin, Hai Bi, Liyuan Tao, Fan Zhang, Hongxian Zhang, Wei Wang, Jitao Wu, Yi Huang, Lulin Ma

**Affiliations:** ^1^ Department of Urology, Peking University Third Hospital, Peking University, Beijing, China; ^2^ Department of Epidemiology, Peking University Third Hospital, Peking University, Beijing, China; ^3^ Department of Urology, Peking Tongren Hospital, Beijing, China; ^4^ Department of Urology, Yantai Yuhuangding Hospital, Yantai, China

**Keywords:** nephrometry score system, renal tumor, laparoscopy, nephron-sparing surgery, “3S+f” nephrometry score system

## Abstract

**Background:**

When we treat renal cell carcinoma by laparoscopic nephron-sparing surgery (NSS), it is essential to use an evaluation system to predict clinical outcomes. Hitherto, there are more than a dozen nephrometry score systems. In this study, through assessing the correlations between nephrometry score systems and clinical outcomes, we aim to provide a novel nephrometry score system—the “3S+f” score system—to simplify the evaluation of technical complexity of partial nephrectomy.

**Methods:**

We retrospectively collected the data of 131 patients who underwent NSS, which was performed by a single surgeon (SZ) from January 2013 to July 2018 at Peking University Third Hospital. The “3S+f” score system contains four parameters: “size, side, site, and fat”, all of which can be obtained from preoperative imaging data. We evaluated the correlations between the “3S+f” score and clinical outcomes, and compared R.E.N.A.L. score and PADUA score.

**Results:**

All the three nephrometry score systems were related to some clinical outcomes in univariate analyses. In multivariate regression models, the “3S+f” score, the R.E.N.A.L. score, and the PADUA score were significantly associated with operative time (*p* = 0.016, *p* = 0.035, and *p* = 0.001, respectively) and warm ischemia time (all *p* = 0.008, *p* < 0.001, and *p* < 0.001, respectively). “3S+f” was also significantly related to extubation time > 5 days (*p* = 0.018). In predicting operative time > 120 min and extubation time >5 days from ROC curves, the AUCs of the “3S+f” score (0.717 and 0.652, respectively) were larger than both the R.E.N.A.L (0.598 and 0.554, respectively) and PADUA (0.600 and 0.542, respectively) score systems.

**Conclusion:**

A novel nephrometry score system—the “3S+f” score system—shows equivalent correlation and the ability in predicting clinical outcomes when compared to the R.E.N.A.L. score system and the PADUA score system, which can describe renal tumors.

## Introduction

The incidence of renal cell carcinoma (RCC) has increased over the last few decades, partly because of the widespread application of imaging, and has become one of the most common malignant tumors in the urinary system today (1, 2). Since the 1960s, Robson et al. have established radical nephrectomy (RN) as the treatment of choice for localized RCC, and it became the gold standard therapy for RCC (3). However, with the increasing early detection of kidney cancer and surgical technology, nephron-sparing surgery (NSS) has attracted the attention of an increasing number of urologists (4, 5). Compared to RN, NSS can provide similar oncologic outcomes and better preservation of renal function in most patients with small renal tumors (6–8). NSS has become the treatment of choice for renal tumors <7 cm (T1a+T1b) according to European Association of Urology guidelines (9).

Different surgical methods involve numerous technical aspects such as tumor exposure, tumor resection, and renal reconstruction. Thus, assessing the surgical anatomy of renal tumors is essential. In 2009, the first system (R.E.N.A.L. nephrometry score) to quantify renal tumor anatomy was developed by Kutikov and Uzzo (10). So far, more than a dozen nephrometry score systems have been developed (11–13). Most of the systems contain several parameters, and the scores can be obtained from preoperative sectional imaging. However, due to their computational complexities, the vast majority of them are hardly applied into practice. Hence, we developed a novel nephrometry score system called “3S+f”, which stands for “size, site, side, and fat”, to quantify the anatomical characteristics of renal masses, aiming to simplify the process of assessment.

In this article, we introduce the “3S+f” nephrometry score system in detail and compare the clinical outcomes with two other common nephrometry score systems: the R.E.N.A.L. (radius, exophytic/endophytic properties, nearness of tumor to collecting system or sinus, anterior/posterior, hilar tumor touching the main renal artery or vein and location relative to polar lines) score system and the PADUA (preoperative aspects and dimensions used for an anatomical classification) score system (10, 14).

## Methods

We collected patients’ data retrospectively *via* the medical record system of the Peking University Third Hospital. To avoid interference from different surgeons, we obtained data of consecutive patients who underwent NSS performed by a single surgeon (SZ) from January 2013 to July 2018. The inclusion criteria for patients were as follows (1): solitary renal mass; (2) laparoscopic nephron-sparing surgery (LNSS); (3) non-solitary kidney; (4) no previous rupture and bleeding of the tumor; and (5) not zero ischemia.

Patients’ epidemiological characteristics included sex, age, height, weight, body mass index (BMI), and comorbidity. Respective parameters and scores of the “3S+f” score system (intrarenal tumor diameter, tumor location, distance to artery/vein/collecting system, and perinephric fat), the R.E.N.A.L. score system (radius, exophytic/endophytic properties, nearness of tumor to collecting system or sinus, anterior/posterior, hilar tumor touching the main renal artery or vein and location relative to polar lines) and the PADUA score system (anterior or posterior face, longitudinal location, rim location; relationships with sinus; relationships with the urinary collecting system; percentage of tumor deepening into the kidney and maximal diameter in centimeters) were defined and calculated according to the described protocols for those systems from preoperative computerized tomography (CT) or magnetic resonance imaging (MRI). American Society of Anesthesiologists (ASA) score and preoperative creatinine (Cr)/hemoglobin (Hb) were also recorded. Clinical outcomes included operative time (OT), warm ischemia time (WIT), estimated blood loss (EBL), reduction in Hb, increase in Cr during operation, postoperative length of stay (PLOS), extubation time (ET), and postoperative complication. Complications were classified according to Clavien–Dindo classification. We also collected information on tumor pathology and T stage as defined by TNM 8th classification (15).

### Nephrometry Score Systems

**Figure 1:**
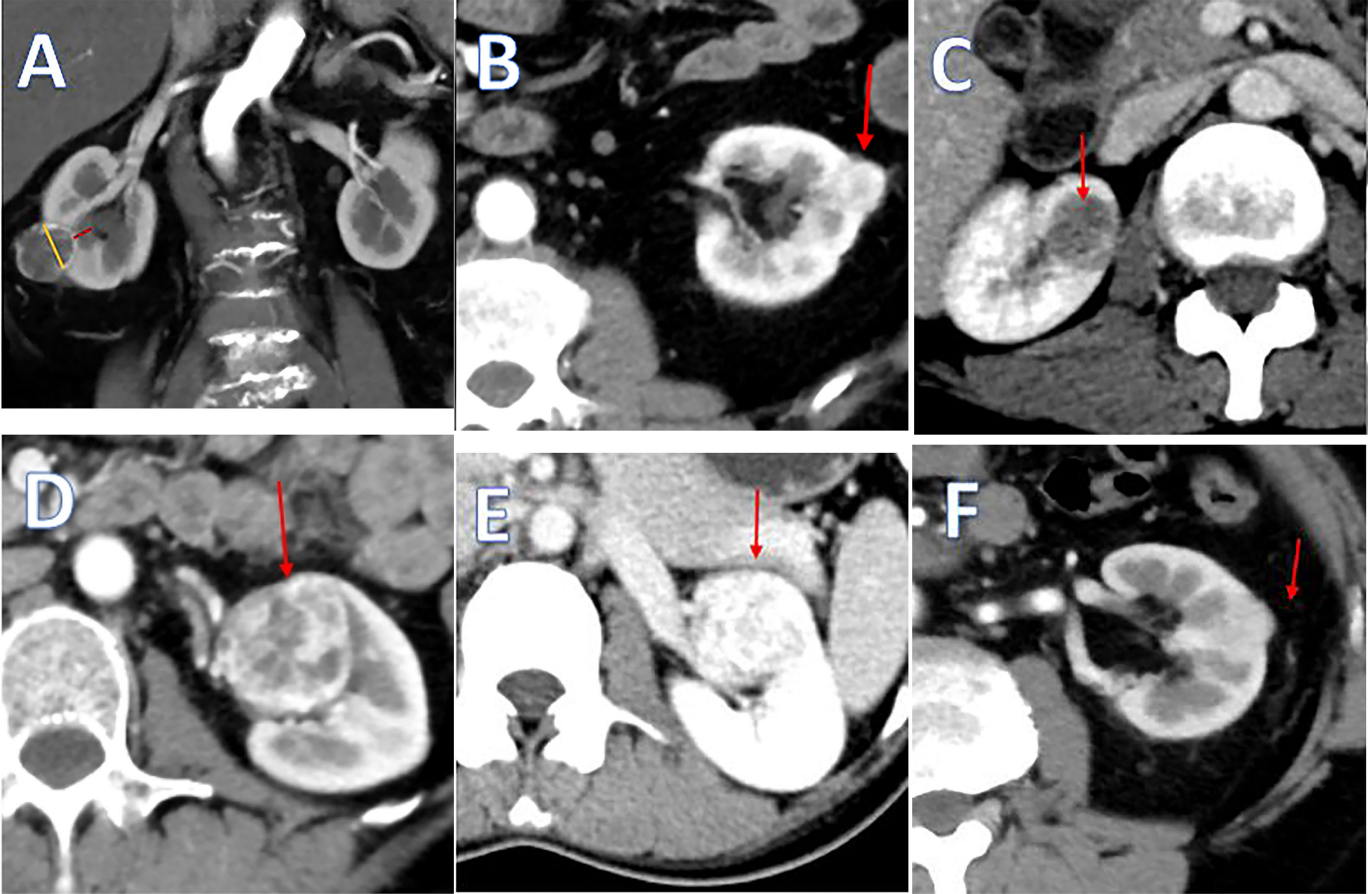
**(A)** The yellow line means the intrarenal diameter of mass, and the red line means distance of the tumor to collecting system. **(B)** Tumor located at the lateral pole. **(C)** Tumor located at the endophytic pole. **(D)** Tumor located at the hilar pole. **(E)** Kidney with thin or hardly any perinephric fat. **(F)** Kidney with thick perinephric fat.

The “3S+f” score consists of four simple separate variables: “size, site, side, and fat”. “Size” refers to the intrarenal diameter of the mass rather than tumor diameter (**Figure 1A**). Actually, after removing the tumor, the volume of renal reconstruction is determined by direct intrarenal tumor volume, indicating the operative complexity to a certain extent. We use the intrarenal diameter of the mass to simplistically represent the intrarenal volume of tumor. “Site” means tumor location (**Figures 1B–D**). In most circumstances, the location of tumor has the greatest correlation with the difficulty of the surgery. Suture is much easier when the tumor is located at the lateral/upper pole, compared to the medial/lower pole. While the renal hilar tumor is close to renal vessels, it is difficult to fix the position of an endophytic tumor, and a cystic tumor is easy to break. Resecting the tumor in these positions is technically challenging. “Side” signifies the distance of the tumor to the artery/vein/collecting system (**Figure 1A**). Many other nephrometry score systems also take this parameter into account. A mass being too close to the artery/vein/collecting system will greatly increase the difficulty of operation. “Fat” denotes the situation of perinephric fat (**Figures 1E, F**). Few nephrometry score systems include this variable. When adhesion exists in perinephric fat, the surgical procedure becomes very complicated and time-consuming. Meanwhile, the renal capsule becomes prone to tearing and bleeding, and could even cause extensive exfoliation of renal capsule and tissues adhering to perinephric fat. Perinephric fat that is sticky or thick as shown in the preoperative CT or MRI will be given 3 points, whereas little or no fat will be given 1 point. Fat classified in between thick fat and little fat will be given 2 points. The detailed scoring rules are shown in **Table 1**.

**Table 1:** “3S+f” nephrometry score system.

3S+f	0pt	1pt	2pt	3pt
**SIZE**	**/**	**<4 cm**	**4–6 cm**	**>6 cm**
**SITE**	**/**	**Lateral/Upper pole**	**Medial/Lower Pole**	**Hilar/Endophytic/Cystic**
**SIDE**	**/**	**>10 mm**	**5–10 mm**	**<5 mm**
**FAT**	**Normal**	**Little/No**	**Some**	**Sticky/Thickness**

The “3S+f” score is further categorized into three groups: low, moderate, and high complexity according to total scores, corresponding to 4–6 points, 7–9 points, and 10–12 points, respectively; the same goes with the R.E.N.A.L. score. For the PADUA score, it is 6–7 points, 8–9 points, and 10–14 points.

### Statistical Analysis

All data analyses were performed by SPSS version 23.0. Spearman correlation analysis was conducted between variables and clinical outcomes. Kruskal–Wallis test was used to evaluate the intergroup distribution differences of three score systems with OT, WIT, EBL, and ET. When finding a significant difference, Bonferroni-adjusted pairwise comparisons were conducted by Mann–Whitney tests. Then, multivariate regression models adjusted for age, sex, comorbidity, BMI, ASA score, and preoperative Cr were constructed to assess the associations between nephrometry score systems’ parameters and clinical outcomes. Multivariable linear regression models were created to evaluate the independent associations between each nephrometry score system and OT or WIT. Clinical outcomes, such as the EBL, ET, and complication, were treated as binary variables, and multivariate binary logistic regression models were designed to assess the abilities of nephrometry score systems’ parameters that predicted these clinical outcomes. According to our hospital’s experience, we defined 100 ml as the demarcation point for EBL and 5 days for ET. Furthermore, receiver operating characteristic (ROC) curves were plotted and areas under the curve (AUCs) were calculated by using a nonparametric distribution assumption for nephrometry score systems to predict OT > 120 min, WIT > 30 min, and ET > 5 days. Statistical significance was set as *p* < 0.05 two-sided.

## Results

A total of 131 consecutive patients were identified and enrolled in our research eventually, whose laparoscopic NSS was performed by the same surgeon (SZ) from January 2013 to July 2018 at Peking University Third Hospital. The demographic and clinical characteristics are summarized in **Table 2**. Among the 131 patients, 60.31% were men and 39.69% were women. The mean age of the patients was 53.60 (18–80), and the mean body mass index (BMI) was 25.30 (16.52–35.91) kg/m^2^. There were 66 (50.38%) patients who had comorbidities such as hypertension, diabetes, smoking history, and chronic renal disease. Sixty-nine (52.67%) patients’ masses were in the left kidney while 62 (47.33%) were in the right. The average ASA score was 1.76. The mean diameter of tumors was 3.07 cm. The mean “3S+f”, PADUA, and R.E.N.A.L. scores were 7.56, 8.95, and 7.30, respectively. For the “3S+f” score, the number of patients classified as low-, moderate-, and high-complexity groups was 22 (16.79%), 100 (76.33%), and 9 (6.87%), respectively. For the R.E.N.A.L. score, the corresponding number of patients was 41 (31.30%), 74 (56.49%), and 16 (12.21%). For the PADUA score, the corresponding number of patients was 30 (22.90%), 46 (35.11%), and 55 (41.98%). The average OT, WIT, EBL, ET, and PLOS were 137.99 min, 22.85 min, 88.24 ml, 4.73 days, and 7.02 days, respectively. The mean increase in Cr and reduction in Hb during operation were 11.20 μmol/L and 14.95 g/L, respectively. Complications occurred in 10 (7.63%) patients, including 5 grade I (3.82%), 4 grade II (3.05%), and 1 grade III (0.76%). Grade I complications included fever, elevation of serum creatinine, and perirenal hematoma without special treatment. Grade II complications were anemia requiring blood transfusion. One grade III complication was renal artery embolization due to artery injury. In 84 (64.12%) patients, the pathological diagnoses were clear-cell carcinoma, 5 (3.82%) were papillary type and 42 (32.06%) were other types. One hundred eleven (84.73%) patients’ surgical margins were negative while 20 (15.27%) were positive. For the pathological T stage, 108 (82.44%) patients were in T1a stage, 21 (16.03%) patients were in T1b stage, and 2 (1.53%) patients were in T2a stage.

**Table 2:** The demographic and clinical characteristics of the patients.

Characteristics		Value (range or *n* %)
Sex		
Male		79 (60.3%)
Female		52 (39.7%)
Age (years)		53.60 (18–80)
BMI (kg/m^2^)		25.30 (16.52–35.91)
Comorbidity		
Yes		66 (50.38%)
No		65 (49.62%)
ASA		1.76
Preoperative Cr (μmol/L)		84.19 (46–328)
Preoperative Hb (g/L)		138.06 (86–168)
Diameter (cm)		3.07 (1.00–9.10)
Location		
Left		69 (52.67%)
Right		62 (47.33%)
3S+f		7.56
Low		22 (16.79%)
Moderate		100 (76.33%)
High		9 (6.87%)
R.E.N.A.L.		7.30
Low		41 (31.30%)
Moderate		74 (56.49%)
High		16 (12.21%)
PADUA		8.95
Low		30 (22.90%)
Moderate		46 (35.11%)
High		55 (41.98%)
OT (min)		137.99
WIT (min)		22.85
EBL (ml)		88.24
ET (days)		4.73
PLOS (day)		7.02
Complication		
No		121 (92.37%)
I		5 (3.82%)
II		4 (3.05%)
III		1 (0.76%)
Increase in Cr (μmol/L)		11.20 (−9–59)
Reduction in Hb (g/L)		14.95 (−21–394)
Histology		
Malignant	Clear cell	84 (64.12%)
	Papillary cell	8 (6.11%)
	Chromophobe cell	5 (3.82%)
	Others	4 (3.05%)
Benign	Angiomyolipoma	11 (8.40%)
	Cyst	10 (7.63%)
	Oncocytoma	5 (3.82%)
	Others	4 (3.05%)
Surgery margin		
Negative		111 (84.73%)
*Positive suspiciously		20 (15.27%)
T stage		
T1a		108 (82.44%)
T1b		21 (16.03%)
T2		2 (1.53%)

ASA, American Society of Anesthesiologists; OT, operative time; WIT, warm ischemia time; EBL, estimated blood loss; ET, extubation time; PLOS, postoperative length of stay; Cr, creatinine; Hb, hemoglobin.

*Positive suspiciously: Cancer cells and suspicious but not certain cancer cells can be seen at the pathological specimen of margins.

The results of Spearman correlation analysis are reported in **Table 3**. According to the statistical results, “size” was significantly related to OT (*p* < 0.001), WIT (*p* = 0.005), and EBL (*p* = 0.001). “Side” was related to reduction in Hb (*p* = 0.017) and ET (*p* = 0.045). “Fat” was related to OT (*p* = 0.023) and EBL (*p* = 0.008).

**Table 3:** Spearman correlation analysis between variables and clinical outcomes.

		OT	WIT	EBL	Reduction in Hb	Increase in Cr	PLOS	ET
Size	Coefficient	0.312	0.242	0.297	0.157	0.015	0.062	0.101
	*p*	<0.001	0.005	0.001	0.074	0.876	0.485	0.252
Site	Coefficient	0.053	0.087	0.021	−0.208	−0.007	−0.017	0.115
	*p*	0.551	0.323	0.812	0.017	0.937	0.844	0.189
Side	Coefficient	0.070	0.351	0.117	0.075	0.132	0.083	0.176
	*p*	0.428	<0.001	0.182	0.396	0.131	0.343	0.045
Fat	Coefficient	0.198	−0.115	0.230	0.080	0.126	0.050	0.070
	*p*	0.023	0.192	0.008	0.365	0.150	0.567	0.935
3S+f	Coefficient	0.279	0.227	0.293	−0.038	0.117	0.081	0.196
	*p*	0.001	0.009	0.001	0.667	0.182	0.355	0.025
R.E.N.A.L.	Coefficient	0.219	0.475	0.184	0.046	0.192	0.043	0.147
	*p*	0.012	<0.001	0.035	0.604	0.028	0.622	0.094
PADUA	Coefficient	0.263	0.487	0.228	0.112	0.131	0.071	0.160
	*p*	0.002	<0.001	0.009	0.205	0.136	0.421	0.068
Age	Coefficient	0.064	−0.052	0.144	0.182	0.024	0.029	0.036
	*p*	0.470	0.557	0.101	0.038	0.787	0.739	0.679
BMI	Coefficient	0.205	0.089	0.190	0.062	0.216	0.090	0.042
	*p*	0.019	0.310	0.030	0.480	0.013	0.309	0.636
ASA	Coefficient	0.141	0.007	0.123	−0.046	0.044	−0.111	−0.062
	*p*	0.109	0.939	0.160	0.602	0.620	0.206	0.479

ASA, American Society of Anesthesiologists; OT, operative time; WIT, warm ischemia time; EBL, estimated blood loss; ET, extubation time; PLOS, postoperative length of stay; Cr, creatinine; Hb, hemoglobin.

The “3S+f” score, R.E.N.A.L. score, and PADUA score were all significantly associated with OT (*p* = 0.001, 0.012, and 0.002, respectively), WIT (*p* = 0.009, *p* < 0.001, and *p* < 0.001, respectively), and EBL (*p* = 0.001, 0.035, and 0.009, respectively). Also, the “3S+f” score was significantly related to ET (*p* = 0.025). However, age, BMI, or ASA score did not evidently correlate with clinical outcomes.

**Table 4** summarizes the results of Kruskal–Wallis test of the different categorized “3S+f” scores’ significant differences in OT (*p* = 0.002), WIT (*p* = 0.002), and EBL (*p* = 0.014). Moreover, the differences between the low- and high-complexity group, and between the moderate- and high-complexity group were remarkably statistically significant with the four clinical variables above. Low- and moderate-complexity groups were not significantly different. There was a correlation between the R.E.N.A.L. score and WIT (*p* < 0.001) but the R.E.N.A.L. score was not related to OT, EBL, or ET. The PADUA score, however, was statistically significantly different with OT (*p* = 0.002), WIT (*p* < 0.001), and EBL (*p* = 0.040).

**Table 4:** Krustal-Wallis test of different nephrometry score systems’ categorized scores.

Variables	I (Low)	II (moderate)	III (high)	H	p	
(median, IQR)						
**3S+f**	n=22	n=100	n=9			
OT (min)	132.50 (102.00-148.25)	128.50 (107.25 -157.00)	196.00 (150.00-243.00)	12.486	0.002	I vs II: no difference; I vs III: p=0.003; II vs III: p=0.002
WIT (min)	17.50 (15.00-24.50)	22.00 (18.75 -27.25)	31.00 (27.00-40.00)	12.173	0.002	I vs II: no difference; I vs III:p=0.002; II vs III: p=0.035
EBL (ml)	20.00 (10.00-87.50)	50.00 (20.00-100.00)	100.00 (100.00-150.00)	8.517	0.014	I vs II: no difference; I vs III: p=0.012; II vs III: p=0.027
ET (day)	5.00 (3.00-5.00)	5.00 (3.00-6.00)	6.00 (5.00-6.00)	4.463	0.107	
**R.E.N.A.L.**	n=41	n=74	n=16			
OT (min)	130.00 (105.00-151.00)	127.00 (108.25-164.00)	142.00 (134.00-159.75)	3.687	0.158	
WIT (min)	17.00 (14.00-22.00)	23.5 (19.00-29.00)	26.50 (22.00-32.50)	25.968	<0.001	I vs II: p<0.001; I vs III: p<0.001; II vs III: no difference
EBL (ml)	20.00 (20.00-100.00)	50.0 (20.00-100.00)	50.0 (20.00-100.00)	4.267	0.118	
ET (day)	7.00 (6.00-8.00)	5.00 (3.25-6.00)	5.00 (3.25-6.25)	1.706	0.426	
**PADUA**	n=30	n=46	n=55			
OT (min)	126.00 (103.50-150.50)	123.00 (101.00-144.00)	146.00 (122.00-183.50)	12.372	0.002	I vs II: no difference; I vs III: p=0.004; II vs III: p=0.032
WIT (min)	18.00 (15.00-21.50)	20.00 (14.25-26.00)	26.00 (22.00-30.50)	30.834	<0.001	I vs II: no difference; I vs III: p<0.001; II vs III: p<0.001
EBL (ml)	20.00 (10.00-87.50)	50.00 (20.00-50.00)	50.00 (20.00-150.00)	6.438	0.04	I vs II: no difference; I vs III: p=0.051; II vs III: p=0.242
ET (day)	4.50 (3.00-6.00)	5.00 (3.00-6.00)	7.00 (6.00-8.00)	2.811	0.245	

OT, operative time; WIT, warm ischemia time; EBL, estimated blood loss; ET, exextubation time.

Furthermore, we analyzed the correlations of different nephrometry score systems for the malignant and benign masses (**Supplementary Table 1**), but there was no correlation between the nephrometry score system and the malignant or benign masses.

To further substantiate the relationships between nephrometry scores and clinical outcomes, we adjusted age, sex, comorbidity, BMI, ASA score, and preoperative Cr to construct the models of multivariable regression analyses. According to **Table 5**, all three nephrometry scores were significantly associated with OT and WIT. For every 1 point increase in the “3S+f” score, an increase in average OT of 7.185 min (95% CI 1.389–12.981, *p* = 0.016) was observed, while the increase was 4.507 min (95% CI 0.319–8.694, *p* = 0.035) in the R.E.N.A.L. score and 7.365 min (95% CI 1.295–2.824, *p* < 0.001) in the PADUA score. Compared with the low-complexity groups, the high-complexity groups’ “3S+f” score (*p* < 0.001) and PADUA score (*p* = 0.006) were statistically significantly related to OT, while this was not found in the R.E.N.A.L. score. The *r* ^2^ values for each scoring system model were 0.136 (“3S+f”), 0.125 (R.E.N.A.L.), and 0.174 (PADUA), indicating that none of these models can explain a high proportion of OT variability.

**Table 5:** Multivariable binary regression analyses of nephrometry score systems and clinical variables.

Variables	OR/Coefficient	95% CI	*p*
OT			
3S+f	7.185	1.389–12.981	0.016
I			Ref
II	1.661	−16.927–29.249	0.860
III	70.668	39.143–102.192	<0.001
R.E.N.A.L.	4.507	0.319–8.694	0.035
I			Ref
II	4.601	−12.525–21.726	0.596
III	22.676	−2.988–48.340	0.083
PADUA	7.365	3.165–11.566	0.001
I			Ref
II	-3.096	−22.226–16.034	0.749
III	27.118	8.075–46.162	0.006
WIT			
3S+f	1.517	0.405–2.628	0.008
I			Ref
II	3.730	−0.007–7.454	0.050
III	11.743	5.428–18.058	<0.001
R.E.N.A.L.	1.995	1.254–2.736	<0.001
I			Ref
II	6.218	3.183–9.253	<0.001
III	10.444	5.896–14.992	<0.001
PADUA	2.059	1.295–2.824	<0.001
I			Ref
II	2.282	−1.229–5.793	0.201
III	8.647	5.152–12.142	<0.001
EBL > 100 ml			
3S+f	1.166	0.812–1.674	0.405
I			Ref
II	0.707	0.220–2.272	0.560
III	3.556	0.603–20.959	0.161
R.E.N.A.L.	1.176	0.908–1.522	0.219
I			Ref
II	0.995	0.357–2.778	0.993
III	2.253	0.556–9.125	0.255
PADUA	1.253	0.960–1.637	0.097
I			Ref
II	0.843	0.224–3.165	0.800
III	2.347	0.707–7.788	0.163
ET > 5 days			
3S+f	1.484	1.071–2.056	0.018
I			Ref
II	2.626	0.802–8.599	0.111
III	10.952	1.736–69.090	0.011
R.E.N.A.L.	1.125	0.906–1.395	0.286
I			Ref
II	1.292	0.542–3.084	0.563
III	1.714	0.485–6.055	0.402
PADUA	1.099	0.883–1.370	0.398
I			Ref
II	1.168	0.423–3.223	0.765
III	1.309	0.477–3.590	0.602
Complication			
3S+f	0.997	0.752–1.322	0.985
I			Ref
II	1.110	0.422–2.917	0.833
III	0.716	0.140–3.659	0.688
R.E.N.A.L.	1.194	0.969–1.471	0.095
I			Ref
II	2.258	0.972–5.246	0.058
III	1.160	0.331–4.061	0.817
PADUA	1.284	1.033–1.596	0.024
I			Ref
II	1.332	0.502–3.539	0.565
III	3.013	1.126–8.063	0.028

OT, operative time; WIT, warm ischemia time; EBL, estimated blood loss; ET, extubation time.

The proportion of WIT explained by each scoring system was also low: *r* ^2^ = 0.035 (“3S+f”), *r* ^2^ = 0.170 (R.E.N.A.L.), and *r* ^2^ = 0.170 (PADUA). For every 1 point increase in the “3S+f” score, there was an increased WIT of 1.517 min (95% CI 0.405–2.628, *p* = 0.008), and the increase was 1.995 min (95% CI 1.254–2.736, *p* < 0.001) in the R.E.N.A.L. score and 2.059 min (95% CI 1.295–1.824, *p* < 0.001) in the PADUA score. Compared with the low-complexity groups, the high complexity groups’ three score systems (all *p* < 0.001) were all significantly related to WIT, and the moderate-complexity groups’ R.E.N.A.L. score system (*p* < 0.001) was significantly correlated with WIT.

**Figure 2:**
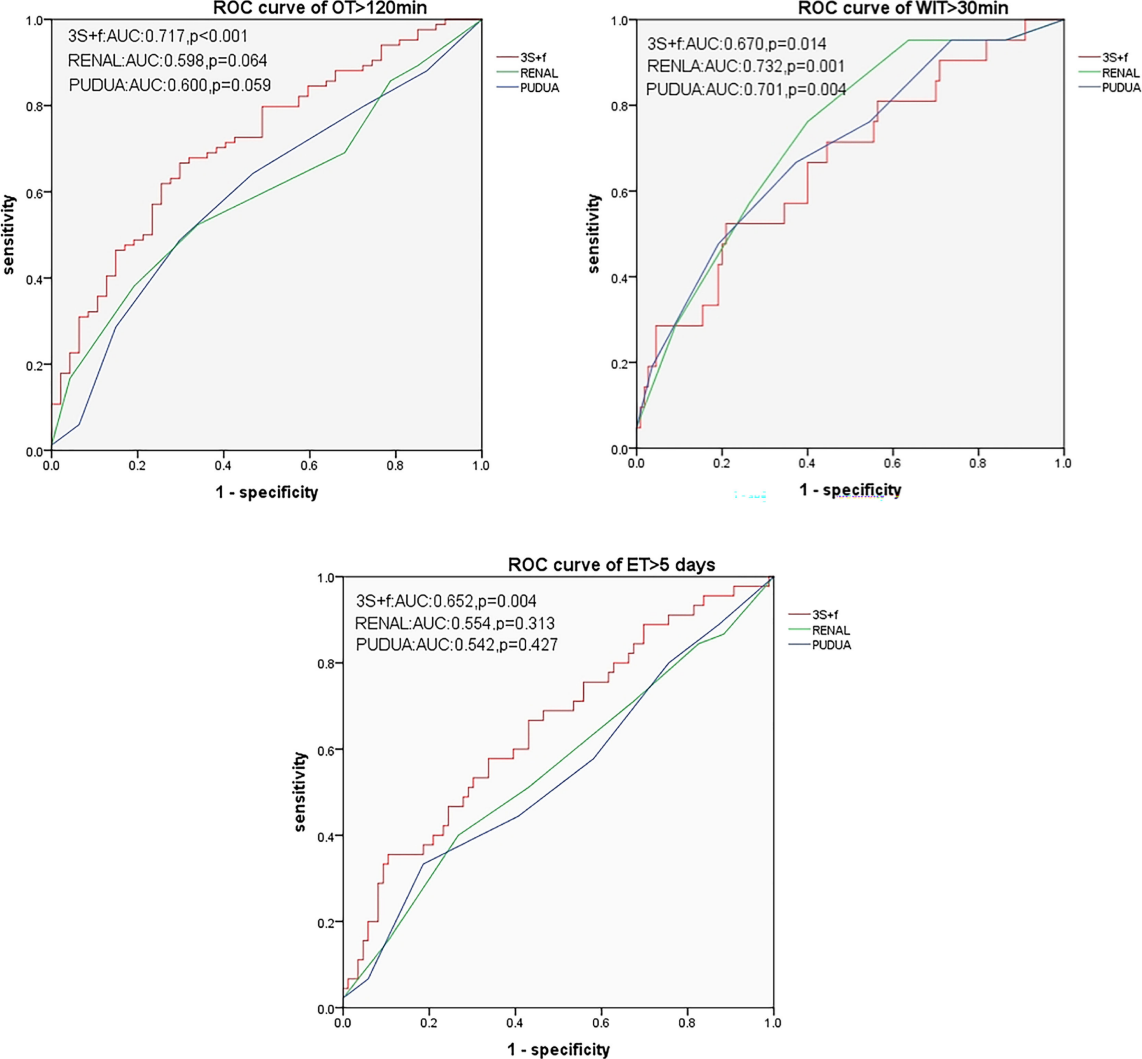
ROC curves for predicting OT > 120 min, WIT > 30 min, and ET > 5 days. OT, operative time; WIT, warm ischemia time; ET, extubation time.

None of these three systems were related to EBL > 100 ml. In predicting OT > 120 min and ET > 5 days, based on ROC curves, the AUCs of the “3S+f” score system (0.717 and 0.652, respectively) were larger than both R.E.N.A.L (0.598 and 0.554, respectively) and PADUA (0.600 and 0.542, respectively) score systems. For predicting WIT > 30 min, the AUC of the “3S+f” score system (0.670) was also nearly equal to R.E.N.A.L (0.732) and PADUA (0.701) score systems (**Figure 2**).

## Discussion

In the treatment of RCC, minimally invasive surgery has been developing rapidly in recent years. The main purposes are to preserve normal renal tissue as much as possible while ensuring complete tumor resection and to reduce perioperative complications. LNSS is now commonly performed to resect solitary kidney tumors. A retrospective study comparing open partial nephrectomy (OPN) with laparoscopic partial nephrectomy (LPN) in the treatment of T1 renal masses found that LPN had less blood loss, shorter surgical duration, and reduced hospital stay (16). Moreover, LPN had similar intraoperative complications (1.8% vs. 1%), similar positive surgical margin rates (1.6% vs. 1%), and similar 3-year oncologic and renal functional outcomes compared to OPN. Although LPN needs more WIT due to its operational difficulty and more postoperative hemorrhage, the advantages of LPN are still obvious. Preoperative assessment of renal mass is essential, but to translate the anatomy of renal tumor into visual data, a standard guide is still lacking, although more than a dozen nephrometry score systems were designed so far. Here, we validate the effectiveness of a novel nephrometry score system called the “3S+f” score system through a single-center, single-surgeon research that included 131 patients who underwent LNSS. We compared our score system with the most commonly used R.E.N.A.L score system and PADUA score system.

OT is a clinical variable we are concerned about. In our study, the total scores of the “3S+f” score system is related to OT (*p* = 0.001) in Spearman correlation analysis. The results of Kruskal–Wallis tests also implied that the higher the “3S+f” score, the more OT would be needed, while the two other score systems simply conveyed a relation of total scores rather than the differences between categorical scores. The multivariable linear regression model and ROC curve also highlighted the fine association between the “3S+f” score and OT, indicating that the “3S+f” score system can be an evidence of length of OT as compared to R.E.N.A.L. and PADUA score systems. As an important clinical factor, usually the more complicated the surgery, the more time it will take. Adequate preoperative preparation can adjust to different nephrometry scores. Surgeons can predict the difficulties caused by a long operative time implied by the “3S+f” score system.

Other studies also assessed the relation between OT and other nephrometry score systems. In a retrospective study of 188 patients undergoing NSS to figure out a nephrometry score system that correlates best with MIC (margin, ischemia, and complications), R.E.N.A.L. and PADUA scores were also found to be associated with OT (17). However, in a study of 162 patients about evaluating the association of tumor size, location, and R.E.N.A.L., PADUA, and centrality index (C-index) scores with perioperative outcomes, R.E.N.A.L. and PADUA scores have not been shown to be significantly related to OT (18). These findings are not in accordance with those of a research on 245 patients investigating R.E.N.A.L and PADUA scores’ clinical significance (19). We believe that OT will be affected by the hospitals’ equipment, skill level of surgeons, and cohort inclusion criteria.

WIT can indicate renal function after operation and is generally considered as the best surrogate for technical complexity (20). At present, it is generally believed that WIT should be within 30 min, and some surgeons take 20 min as the upper limit (21).

Our patients had a mean WIT of 22.85 min, 21 of whom had a WIT of more than 30 min. All the three scores were significantly associated with WIT no matter what statistical method was used. For every 1 score increase, there was an increased WIT of 1.517 min (95% CI 0.405–2.628, *p* = 0.008) in the “3S+f” score, 1.995 min (95% CI 1.254–2.736, *p* < 0.001) in the R.E.N.A.L. score, and 2.059 min (95% CI 1.295–2.824, *p* < 0.001) in the PADUA score. We can conclude that with more scores of the three systems, more WIT will need longer vascular clamp time to ensure the patient’s safety during tumor removal. The results were also illustrated by some other studies. Okhunov et al. designed a 101-patient retrospective study to establish reliability and assess the relationships between R.E.N.A.L., PADUA, and C-index scores, and perioperative and postoperative variables, and they found that all the three scores were significantly associated with WIT (C-index, *p* < 0.001; PADUA, *p* = 0.016; R.E.N.A.L., *p* < 0.001) and percent change in creatinine level (C-index, *p* < 0.001; PADUA, *p* < 0.001; R.E.N.A.L., *p* < 0.001) (20). In our ROC curves, PADUA had a stronger ability to predict OT > 30 min. In fact, in a series of studies, PADUA score was considered significantly related to WIT due to its detailed anatomical parameters (22–24). We did not emphasize the renal function in our study since no parameter was related with an increase in Cr in Spearman correlation analysis, but renal function was still related to nephrometry scores, which was approved by other researchers. Matthew et al. found that R.E.N.A.L. and C-index scores were associated with percent functional volume preservation (each *p* < 0.001) and were also related with nadir and late percent glomerular filtration rate preservation in a 237-patient study (25). A study from Taiwan indicated that three scoring systems performed significant correlation with both early and late functional outcomes (*p* < 0.05), and R.E.N.A.L. nephrometry and PADUA classification reported high correlation (−0.715 and −0.721), while C-index demonstrated moderate correlation (0.451) (26).

Evaluation of surgical complications is of great importance when evaluating outcomes of surgery. Although NSS is as effective as RN in long-term oncologic outcome, patients who underwent PN are at a higher risk of postoperative complications compared with those who underwent RN, especially for intrarenal tumors (6, 27–29). LNSS with more WIT increases the incidence of complications. However, we did not obtain results confirming that these three systems were associated with complications from our study. A novel anatomic classification system called Zhongshan score with R.E.N.A.L. and PADUA scores had a definite correlation with severe complications and surgical complications in a retrospective study of 789 patients who underwent NSS (13). In another study that assesses a zero ischemia index (ZII), R.E.N.A.L. and PADUA scores in predicting complexity, and outcomes of off-clamp partial nephrectomy, results showed that ZII (*p* = 0.020), R.E.N.A.L. (*p* = 0.014), and PADUA (*p* = 0.027) scores were all related to surgical complications (12). Results of complication are also heterogeneous among different studies. A study that compared four established nephrometry systems—R.E.N.A.L., PADUA, NePhRO, and C-index—for their significance in predicting the surgical outcome of partial nephrectomy in a cohort of 305 patients found that none of them was related to severe complications (30). With the increasing LPN experience of surgeons and more common use of techniques to reduce WIT (e.g., early unclamping, off-clamp partial nephrectomy, selective arterial clamping, and parenchymal clamping), we believe that improvement will be made in this issue.

We also pay attention to PLOS and ET, which indicate patients’ surgical damage and physical recovery to a certain degree. Also, PLOS and ET are associated with patients’ expense that patients care about. Some studies may add LOS to their study, but no one has assessed ET as far as we are concerned (17). Although PLOS was not related to any variable in Spearman correlation analysis, we found that “3S+f” had high correlation (*p* = 0.025) with ET and a great ability (*p* = 0.018) to predict ET > 5 days. Every one score increase in the “3S+f” score system was associated with a 1.484-fold high risk of tending to ET > 5 days, which elucidated that more attention need to be paid on the drainage tube (used to remove blood or other fluids from the surgical wound) to care for patients who scored higher in the “3S+f” score system. We predicted that the extubation time may be better predicted with “3S+f” as it takes perinephric fat into consideration, which may be related to the degree of adhesion of the surgical areas.

Besides these evaluations of perioperative outcomes, we take the histology of the tumors into consideration as well, but found no association between each of the three nephrometry scores and the presence of malignancy. In fact, in addition to gender (female patients have more benign tumors) and tumor size (larger size correlated with malignancy), few tumor anatomic characteristics or patient features were found to be significant predictors for differentiating malignant and benign renal masses (31).

We have chosen four parameters as components of the “3S+f” score system, namely, “size, site, side, and fat”, and any of them contributes to the complexity of surgery in theory. The “3S+f” score system has only four parameters and all can be easily calculated from preoperative images. In this study, we chose patients who only underwent LNSS to eliminate the negative effect from different surgical methods. All surgeries were performed by the same surgeon (SZ), which is distinctive, and which many other studies failed to achieve. With only one surgeon, many biases can be eliminated. However, there are several limitations in our study. A small sample (131 patients) may not be enough to assess our system. The lack of different raters can also lead to biases. In addition, we only collected data from a single center. Also, our retrospective study may be affected by subjective factors when we reviewed the patients’ data. Furthermore, the assessment of SITE and FAT may suffer from subjective bias since these two indicators can hardly be quantified by using a rigorous objective method. We also found that “3S+f” and PADUA only differentiate tumors with low vs. high complexity and moderate vs. high complexity but not between tumors with low vs. moderate complexity. The same results can be demonstrated by the study of Okhunov et al., although their results revealed that nephrometry scores (R.E.N.A.L., PADUA, and C-index) could only differentiate tumors with low vs. moderate/high complexity but not between tumors with moderate vs. high complexity, which suggested that a two-tiered complexity classification may be more valid (20). Therefore, further exploration is required to validate the effectiveness of the “3S+f” score system in the future.

## Conclusions

Our novel nephrometry score system—the “3S+f” score system—has simple components and is easy to calculate from preoperative cross-sectional imaging. Compared with R.E.N.A.L. and PADUA scores, it shows equivalent correlation and the ability to determine clinical outcomes, demonstrating that our system is reliable, to some degree, in predicting surgical complexity. Thus, we provide a new nephrometry score system to describe renal tumors suitable for NSS.

## Data Availability Statement

The raw data supporting the conclusions of this article will be made available by the authors, without undue reservation.

## Author Contributions

SZ, ZQ, and LM contributed to the study design and administrative support, and were major contributors to the writing of the manuscript. SZ, ZQ, FZ, HZ, YH, and LM provided the study materials and patients. SZ, ZQ, LM, and LT were in charge of data analysis and interpretation. All authors contributed to data collection, manuscript writing, and final approval of the manuscript.

## Funding

This work was supported by the National Natural Science Foundation of China (General Program, 82072828); the Special Fund for Clinical Research of Wu Jieping Medical Foundation (320.6750.2020-13-2); Beijing Municipal Health Commission, Hygiene and Health Science and Technology Achievements and Appropriate Technology Promotion Program (BHTPP202056); Clinical Cohort Construction Projects of Peking University Third Hospital (BYSYDL2019010) and Key Clinical Projects of Peking University Third Hospital (BYSYZD2019016).

## Conflict of Interest

The authors declare that the research was conducted in the absence of any commercial or financial relationships that could be construed as a potential conflict of interest.

## Publisher’s Note

All claims expressed in this article are solely those of the authors and do not necessarily represent those of their affiliated organizations, or those of the publisher, the editors and the reviewers. Any product that may be evaluated in this article, or claim that may be made by its manufacturer, is not guaranteed or endorsed by the publisher.

## Abbreviations

ASA, American Society of Anesthesiologists; AUC, area under the curve; AUCs, areas under the curve; BMI, body mass index; CT, computerized tomography; EBL, estimated blood loss; ET, extubation time; LNSS, laparoscopic partial nephrectomy; LOS, length of stay; LPN, laparoscopic partial nephrectomy; MRI, magnetic resonance imaging; NSS, nephron-sparing surgery; OPN, open partial nephrectomy; OT, operative time; PLOS, postoperative length of stay; RCC, renal cell carcinoma; RN, radical nephrectomy; ROC, receiver operating characteristic; SZ, Shudong Zhang; WIT, warm ischemia time.
