# Supplementary material for: A “3S+f” Nephrometry Score System to Predict the Clinical Outcomes of Laparoscopic Nephron-Sparing Surgery
Source: Front Oncol. 2022 Jul 14;12:922082. doi: 10.3389/fonc.2022.922082 (PMC9330399; doi:10.3389/fonc.2022.922082)
Supplement: Supplementary file 1 [file Table_1.docx]

Supplemental Table 1: Univariate analysis of nephrometry score systems for malignant and benign masses

| Variables | OR | 95%CI | p |
| --- | --- | --- | --- |
| 3S+f | 1.122 | 0.826-1.524 | 0.461 |
| I |  |  | ref |
| II | 0.972 | 0.241-3.928 | 0.968 |
| III | 2.549 | 0.758-8.570 | 0.130 |
| RENAL | 1.208 | 0.967-1.510 | 0.096 |
| I |  |  | ref |
| II | 0.345 | 0.068-1.757 | 0.200 |
| III | 0.518 | 0.106-2.518 | 0.415 |
| PADUA | 1.171 | 0.930-1.476 | 0.180 |
| I |  |  | ref |
| II | 0.468 | 0.155-1.410 | 0.177 |
| III | 0.389 | 0.146-1.034 | 0.058 |
